# Mediating effects of attention problems on the link between parenting style and internet gaming disorder in adolescents

**DOI:** 10.3389/fpsyt.2023.1211889

**Published:** 2023-07-27

**Authors:** Sung Ah Chung, Sujin Bae, Hee Jin Kim, Jea Woog Lee, Hyunchan Hwang, Doug Hyun Han

**Affiliations:** ^1^Department of Psychiatry, Chung-Ang University Hospital, Seoul, Republic of Korea; ^2^Department of Information and Technology in Sport, College of Sports Science, Chung-Ang University, Anseong-si, Republic of Korea

**Keywords:** internet gaming disorder, attention deficit hyperactivity disorder, parenting style, mediation effect, gaming pattern

## Abstract

**Background:**

Positive and negative parenting styles as well as psychiatric comorbidities including attention deficit hyperactivity disorder (ADHD) have been associated with internet gaming disorder (IGD) in children and adolescents. We hypothesized that ADHD and parenting style would be associated with IGD in adolescents. In addition, psychological status could mediate the link between parenting style and the severity of IGD.

**Methods:**

A total of 256 adolescents with IGD and 211 healthy internet game players and their mothers participated in the current study. Demographic data, gaming patterns, and psychological status including ADHD were recorded for all adolescents. The parenting style of each adolescent’s mother was assessed using the Maternal Behavior Research Instrument (Korean version).

**Results:**

There were significant differences in the internet game play patterns, psychological status, and parenting styles between the IGD group and healthy internet game players. In the hierarchical logistic regression analysis, higher ADHD scores, less affective parenting styles, and less autonomous parenting styles, were significant predictors of IGD. In the mediation test, the ADHD score was found to mediate the association between affective and autonomous parenting styles and the severity of IGD.

**Conclusion:**

Attention problems could directly and indirectly mediate the relationship between positive parenting styles and the severity of IGD. Our findings have the potential to aid in the development of treatment plans for IGD and ADHD as well as to contribute to the development of educational resources regarding parenting styles.

## Introduction

### Parenting style and child and adolescent behavior

Parenting style is defined as the manner in which parents rear their children (1–3). Parenting behaviors are known to be associated with adolescents’ emotional and problem behaviors (1, 2). Schaefer et al. (3) suggested two independent parenting styles of emotion and control. The emotion dimension involves parents’ emotional attitude toward the child (affection vs. rejection), while the control dimension involves parents’ exerting control over the child’s behavior (autonomy vs. control) (3). According to Baumrind (4), with the combination of these two dimensions, four parenting styles can be derived: authoritative (highly supportive and flexible), authoritarian (lack of support and rigid control), permissive (lack of control), and neglecting (lack of support and control). Each parenting style is associated with children’s behavioral patterns and symptoms, and these are related to various socialization processes and emotional aspects. The authoritative parenting style is associated with positive outcomes including emotional adjustment and healthy self-esteem as well as low aggression, anxiety, and depression (5–7). In contrast, the other three styles are associated with negative outcomes including emotional lability, low sociality, and unstable attachment (7, 8). Children of authoritarian parents commonly exhibit withdrawn and hostile behavior. Children of permissive parents often encounter difficulties in regulating their behaviors and emotions and may struggle academically due to the low level of parental control. However, they tend to possess positive self-perception and demonstrate relatively lower levels of depressive symptoms. Finally, the neglecting parenting style is associated with the poorest outcomes in various developmental, behavioral, emotional, and social aspects in children. They often feel emotionally detached and report the highest levels of depression (4). While the authoritative parenting style is typically associated with positive outcomes, these findings do not universally apply to all of society. Gonzalez et al. (9) reported that in African-American college students, the authoritarian parenting style was related to seeking challenges and being competent. Similarly, McBride-Chang and Chang (10) reported that in students in Hong Kong, the authoritative parenting style was actually associated with negative outcomes such as low emotional autonomy.

### Parenting style and adolescent with internet gaming disorder

Several studies have suggested that parent–child relational problems in the family are related to internet use patterns in adolescents (11–14). Trumello et al. (14) reported that excessive internet use was negatively associated with maternal care. Mother’s avoidance and father’s anxiety were also found to be directly associated with problematic internet use in adolescents (13). Furthermore, in a longitudinal study of 1,153 Taiwanese students, low family support, a less protective parenting style, and high attention deficit hyperactivity disorder (ADHD)-related symptoms were associated with problematic internet use (12). In addition, Arikan et al. (11) suggested that maternal attachment anxiety indirectly predicted the overuse of a social network service (SNS) in young adults via young adult attachment anxiety.

Among popular internet-related activities, internet game playing is also known to be associated with family functions and parenting style (15–18). In a study involving 682 Iranian adolescents, parenting style was significantly associated with the prevalence of internet gaming disorder (IGD) (15). A dominant permissive parenting style was associated with symptoms of IGD in children (17). Bonnaire and Phan (16) suggested that parental close monitoring the process of an adolescent’s internet use activity could decrease the risk of developing IGD. Throuvala et al. (18) reported that parental rejection could predict IGD through the mediating effect of core self-evaluation in adolescents.

### Parenting style and adolescents with attention deficit hyperactivity disorder

ADHD is a prevalent mental disorder that impacts approximately 3 to 5% of students and is characterized by symptoms such as hyperactivity, inattention, and impulsive actions (19). Previous studies have shown that parents of children with ADHD are less authoritative and show an authoritarian parenting style. This parenting style is characterized by high expectations and strict rules set by the parents, and verbal communication is usually one-sided and lacks an emotional response. This can cause their children to feel isolated and depressed and increase their negative symptoms of ADHD (20).

Previous studies have also shown that the parents of children with ADHD have a greater tendency to use punishment to control children with hyperactivity than other parents, meaning they employ a more authoritarian parenting style. On the other hand, permissive parenting styles were lower than other parents (4, 21, 22).

### Psychological status and adolescents with IGD

Many studies of IGD have suggested that comorbidities play a critical role in the development of IGD (23–26). In a cohort of 755 patients with IGD, Han et al. (24) suggested that age, family support, social factors, and psychological status including attention, anxiety, and mood could impact the initial and sustained responses to treatment for IGD. Ko et al. (25) suggested that psychiatric diseases including ADHD, major depressive disorder (MDD), and social anxiety disorder were significantly associated with the prevalence of IGD. Lee et al. (26) reported that a comorbidity of ADHD in patients with IGD was associated with a poor clinical course in response to treatment. Furthermore, Cudo et al. (23) suggested that depression could mediate the correlation between self-esteem and IGD.

Remondi et al. (27) also suggested that insecure attachment between adolescents and their parents had an indirect effect on the development of problematic use of mobile devices (smartphones and tablets) mediated by psychological risk factors. Similar to the findings of Remondi et al. (27), we speculated that psychological status could mediate the association between parenting style and the severityof IGD.

### Hypothesis

We hypothesized that ADHD and parenting style would be associated with IGD in adolescents and that affective parenting styles would have particularly positive effects on IGD. In addition, psychological status could mediate the link between parenting style and the severity of IGD.

## Methods

### Participants

From August 2016 to July 2020, patients who visited the IT and Human Research Center at Chung Ang University Hospital for the treatment of IGD were asked to participate and recruited in the current study. The inclusion criteria were as follows: (1) diagnosis of IGD based on the Diagnostic and Statistical Manual of Mental Disorders (DSM)-5 (28); (2) adolescents aged from 13–18 years; and (3) adolescents living with their mothers. The exclusion criteria were as follows: (1) history of a psychotic disorder including bipolar disorder, schizophrenia, or severe depressive disorder with psychotic features; (2) history of developmental disorders including autism spectrum disorder (ASD) or intellectual disability (intelligence quotient (IQ) < 70); and (3) history of chronic medical conditions.

Of 374 consecutive patients who were eligible to participate in the current study, 295 patients and their mothers agreed to take part. Of 295 patients, 19 patients were excluded due to low intelligence, 11 patients were excluded due to ASD, five patients were excluded due to bipolar disorder, and four patients were excluded due to schizophrenia. Finally, 256 adolescents with IGD and their mothers participated in the research.

As a comparison group, healthy internet game players were recruited from the same hospital via flyers and banner advertisements. The inclusion criteria were as follows: (1) adolescents who played internet games for at least 2 hours per week [the mean game play time in Korean adolescents has been to be 2.5 h per week (Game Self-Governance Organization of Korea, GSOK, http://www.gsok.or.kr/gsok-news/?mod=document&uid=1914)]; (2) adolescents aged from 13–18 years; and (3) adolescents living with their mothers. The exclusion criteria were follows: (1) diagnosis of IGD based on the DSM-5 (28); (2) history of a psychotic disorder including bipolar disorder, schizophrenia, or severe depressive disorder with psychotic features; (3) history of developmental disorders including ASD or intellectual disability (IQ < 70); and (4) history of chronic medical conditions.

Of 233 individuals who wanted to participate in the current study, 211 healthy participants were recruited. Eleven participants were excluded due to a diagnosis of IGD. Five participants were excluded due to low intelligence. Three participants were excluded due to bipolar disorder. Three participants were excluded due to ASD.

All participants understood the study procedures and agreed to participate voluntarily in the current study. The current research received approval from the Chung Ang University Institutional Review Board (1990-007-386), and the participants completed and signed consent forms. Adolescents also provided written consent for participation from their parents or guardians. The study was conducted in accordance with the Declaration of Helsinki.

### Measures

Demographic data were assessed which could affect bias. The severity of IGD was assessed using Young Internet Addiction Scale Korean version (29, 30). This scale consists of 20 items that measure problematic internet use including internet games and has good internal consistency (Cronbach’s *α* = 0.90). The cutoff values of the IAT were as follows: <20: below average users; 20–49: average users; 50–79: occasional/frequent problems; and 80–100: significant problems (29, 30).

Depressive mood was assessed using the Beck Depression Inventory II (BDI-II) (31), which consists of 21 self-report items that are rated on a 4-point Likert-type scale ranging from 0 to 3. The total BDI-II score can range from 0 to 63. The anxiety level was assessed using the Beck Anxiety Inventory (BAI) (32), which consists of 21 self-report items that are rated on a 4-point Likert-type scale ranging from 0 to 3. The total BAI score can range from 0 to 63. Both the BDI-II (Cronbach’s *α* = 0.89) (33) and BAI (Cronbach’s alpha = 0.95) (32) have good internal consistency.

IQ was estimated using the Korean Wechsler Intelligence Scale for Children IV (K-WISC-IV), which was administered by clinical psychologists (34) The K-WISC-IV is modified from WISC-IV and designed for persons aged from 6 years to 16 years 11 months. It consists of four sub-index scores: the Verbal Comprehension Index, Perceptual Reasoning Index, Working Memory Index, and Processing Speed Index. IQ assessment was conducted by clinical psychologists in the same hospital, Chung-Ang University Hospital.

Attention problems were assessed using the Korean version of Dupaul’s ADHD Rating Scale (K-ARS) (35, 36), which includes 18 items including 9 items for assessing inattention and 9 items for assessing hyperactivity. The internal consistency of the K-ARS ranges from 0.77 to 0.89 (36). Behavioral control was assessed using the Behavioral Inhibitory System/Behavioral Activation System (BIS/BAS) Scale (37, 38). The BIS/BAS is composed of a four-point Likert scale with responses ranging from “not at all” to “strongly agree.” The total scores of the BIS/BAS range from 0 to 80. The internal consistency (Cronbach’s *α*) of the BIS/BAS has been reported to range from 0.78 to 0.79 (38).

The parenting style of each adolescent’s mother was assessed using the Maternal Behavior Research Instrument (MBRI-K) (Korean version) (3, 39). The MBRI-K is a 48-item, self-report instrument assessing maternal parenting attitudes. Parenting styles were classified into four types including affective, rejecting, autonomous, and controlling types. Each style has 12 items and is scored by summing the subscale responses (values ranging from 1 to 5). The sum of the subscale scores ranges between 12 and 60. Higher scores on each subscale indicate that the maternal parenting attitude shows a greater degree of that style.

### Statistical analysis

The differences in demographic data including age, sex, education level, and smoking and alcohol habits between the IGD and healthy game play groups were analyzed using independent *t*-tests and the chi-square test. The differences in game play style including the genre of game play, mean game play time in a weekday, and mean game play time on the weekend between the IGD and healthy game play groups were analyzed using the chi-square test and independent *t*-tests. The differences in psychological scale scores including the YIAS, BDI-II, BAI, K-ARS, BISBAS, and KWAIS scores as well as the differences in parenting styles between the IGD and healthy game play groups were analyzed using independent *t*-tests.

The Durbin-Watson test was used to confirm the problem of collinearity of the data. Hierarchical logistic regression was used to assess how much the variables in the questionnaires, psychological scales, and parenting styles explained a statistically significant amount of the variance in the dependent variable of IGD. In a multiple hierarchical regression analysis of all participants, a discrete set of hierarchical variables, with IGD as the dependent variable, was added: demographic factors were included in model 1; model 1 factors + game play patterns were included in model 2; model 2 factors + psychological state were included in model 3; and model 3 factors + parenting style were included in model 4. The dependent variable of “IGD” was coded as “1,” and the healthy game players were coded as “0.” The definition of “IGD” coincided with the inclusion criteria above.

The mediating role of attention problems on the relationship between parenting style and IGD was assessed using Hayes’s PROCESS macro for SPSS (model 4) (40). The mediating effect verification model was divided into two models according to the factors related to the parenting method. The independent variable of Model A was set as a parent effective variable, and the independent variable of Model B was set as a parent autonomous variable. In addition, the dependent variable was set as the YIAS score, and the K-ARS score was set as a parameter to verify the direct and indirect effects of ADHD on the YIAS score through the parenting style. With the bootstrap confidential intervals (CIs) in PROCESS, the indirect effect was considered significant if the 95% CI did not include zero.

## Results

### Demographic data, game play pattern, psychological status, and parenting style

A total of 467 participants’ data were analyzed. There were no significant differences in age, sex distribution, education level, or smoking and alcohol habits between the IGD and healthy game play groups (Table 1).

**Table 1:** Demographic data, psychological characteristics, and parenting style.

	IGD group	Healthy game play group	Statistics
Demographic information			
Age	15.7 ± 2.0	15.5 ± 1.9	*t* = 1.10, *p* = 0.27
Sex (male/female)	239 (93.4%)/17 (6.6%)	194 (91.9%)/17 (8.1%)	*χ*^2^ = 0.34, *p* = 0.59
Education level (years)	8.8 ± 2.0	8.6 ± 1.9	*t* = 1.00, *p* = 0.32
Smoking			
Heavy	10 (3.9%)	13 (6.2%)	*χ*^2^ = 1.37, *p* = 0.51
Occasional	35 (13.7%)	26 (12.3%)	
Non-smoking	211 (82.4%)	172 (81.5%)	
Alcohol			
Heavy	5 (2.0%)	5 (2.4%)	*χ*^2^ = 1.56, *p* = 0.46
Occasional	38 (14.8%)	40 (19.0%)	
Non-drinking	213 (83.2%)	166 (78.7%)	
Game play style			
Game genre			
MMORPG	87 (34.0%)	85 (40.3%)	*χ*^2^ = 2.03, *p* = 0.57
RTS	112 (43.8%)	85 (40.3%)	
FPS	30 (11.7%)	21 (10.0%)	
Others	27 (10.5%)	20 (9.5%)	
Play time each weekday (hours/day)	4.1 ± 3.1	2.6 ± 1.9	*t* = 6.42, *p* < 0.001*
Play time each weekend (hours/day)	4.6 ± 3.3	2.9 ± 1.8	*t* = 6.99, *p* < 0.001*
Psychological scales			
YIAS	57.3 ± 13.7	34.2 ± 12.5	*t* = 18.87, *p* < 0.001*
BDI-II	16.3 ± 10.9	10.9 ± 8.5	*t* = 5.86, *p* < 0.001*
BAI	11.6 ± 9.4	8.6 ± 7.7	*t* = 3.71, *p* < 0.001*
K-ARS	16.8 ± 12.9	12.6 ± 12.0	*t* = 3.61, *p* < 0.001*
BISBAS	55.4 ± 8.1	51.2 ± 8.9	*t* = 5.41, *p* < 0.001*
K-WAIS total	101.7 ± 18.2	103.2 ± 18.5	*t* = −0.86, *p* = 0.39
Parenting style			
Affective attitudes	34.3 ± 7.5	38.5 ± 7.0	*t* = −6.23, *p* < 0.001*
Rejecting attitudes	37.6 ± 7.9	38.4 ± 7.1	*t* = −1.09, *p* = 0.28
Autonomous attitudes	33.5 ± 6.8	35.7 ± 7.3	*t* = −3.29, *p* = 0.001*
Controlling attitudes	36.5 ± 5.6	37.6 ± 6.0	*t* = −2.05, *p* = 0.04

K-ARS, Korean version of Dupaul’s ADHD rating scale; BIS/BAS, behavioral inhibitory system/behavioral activation system; K-WAIS-IV, Korean–Wechsler adult intelligence scale-IV; MMORPG, massively multiplayer online role-playing games; FPS, first-person shooter; RTS, real-time strategy; YIAS, young internet addiction scale; BDI-II, beck depression inventory II; BAI, beck anxiety inventory. Hierarchical logistic regression analysis.*p < 0.001.

There was no significant difference in the game genre distribution between the IGD and healthy game play groups. However, those in the IGD group played internet games more times on weekdays and weekends than those in the healthy game play group. The IGD group showed increased YIAS scores compared with the healthy game play group.

The IGD group showed increased scores on the BDI-II, BAI, K-ARS, and BISBAS compared with the healthy game play group (Table 1). There was significant difference in the prevalence of a comorbidity of ADHD between the IGD and healthy game play groups (*χ*^2^ = 103.7, *p* < 0.01). Of 256 individuals with IGD, 165 (64.5%) had a comorbidity of ADHD; in contrast, only 37(17.5%) of 211 healthy game players had a comorbidity of ADHD. However, there was no significant difference in the K-WAIS total scores between the IGD and healthy game play groups.

The scores for maternal affective attitudes and autonomic attitudes in the IGD group were significantly decreased compared with those observed in the healthy game play group (Table 1). The scores for controlling attitudes in the IGD group were decreased compared with those observed in the healthy game play group. There was no significant difference in the scores for controlling attitudes between the two groups.

Considering the results of the Durbin–Watson test, there was no autocorrelation in the current dataset. Of the four models suggested in the current study, models 2, 3, and 4 were significantly associated with IGD. In model 2, the model *χ*^2^ (67.8, *p* < 0.01) and Nagelkerke’s R2 (0.181, 18.1% of the variance in the dependent variable of IGD) indicated that the model was adequate to predict IGD. When the practical usefulness of the model was assessed considering the classification accuracy, eight variables in model 2 enhanced the predictive accuracy of the group membership of the dependent variable to 65.7%. With the step *χ*^2^-value (Step *χ*^2^ = 64.1, *p* < 0.01), game play pattern was found to be a predictive factor for IGD.

In model 3, the model *χ*^2^ (109.7, *p* < 0.01) and Nagelkerke’s R2 (0.280, 28.0% of the variance in the dependent variable of IGD) indicated that the model was adequate to predict IGD. When the practical usefulness of the model was assessed considering the classification accuracy, 13 variables in model 3 enhanced the predictive accuracy of the group membership of the dependent variable to 70.2%. With the step *χ*^2^-value (Step *χ*^2^ = 41.9, *p* < 0.01), psychological status was a predictive factor for IGD.

In model 4, the model *χ*^2^ (147.8, *p* < 0.01) and Nagelkerke’s R2 (0.363, 36.3% of the variance in the dependent variable of IGD) indicated that the model was adequate to predict IGD. When the practical usefulness of the model was assessed considering the classification accuracy, 17 variables in model 4 enhanced the predictive accuracy of the group membership of the dependent variable to 72.8%. With the step *χ*^2^-value (Step *χ*^2^ = 38.0, *p* < 0.01), parenting style was a predictive factor for IGD.

According to the Wald statistics for all independent variables, higher ADHD scores, less affective parenting styles, and less autonomous parenting styles were significant predictors of IGD (Table 2).

**Table 2:** Hierarchical logistic regression analysis.

Independent variables		Model 1			Model 2			Model 3			Model 4		
		B	Wald	OR	B	Wald	OR	B	Wald	OR	B	Wald	OR
Demographic characteristics	Sex	−0.193	0.289	0.824	−0.196	0.266	0.822	−0.652	2.597	0.521	−0.681	2.319	0.506
	Age	0.376	0.857	1.457	0.620	2.038	1.859	0.655	2.069	1.926	0.665	1.965	1.944
	Education	−0.316	0.603	0.729	−0.568	1.716	0.567	−0.662	2.117	0.516	−0.654	1.901	0.520
	Smoking	−0.254	0.141	0.775	−0.524	0.469	0.592	−0.675	0.680	0.509	−0.538	0.379	0.584
	alcohol	−0.418	2.003	0.658	−0.371	1.344	0.690	−0.257	0.599	0.774	−0.274	0.609	0.760
Game play pattern	Game genre				0.143	1.718	1.153	0.114	1.015	1.121	0.136	1.329	1.145
	Weekday				0.165	9.327	1.179**	0.142	6.389	1.153*	0.100	2.658	1.106
	Weekend				0.175	9.388	1.239**	0.139	5.103	1.125*	0.138	1.401	1.148
Psychological status	BDI-II							0.007	0.572	1.007	0.002	0.018	1.002
	BAI							−0.009	0.322	0.991	0.003	0.021	1.003
	KARS							0.047	10.389	1.049**	0.049	9.805	1.050**
	BISBAS							0.054	15.751	1.055	0.001	0.004	1.001
	K-WAIS							−0.002	0.106	0.998	0.002	0.077	1.002
Parenting style	Affective										−0.217	26.578	0.717**
	Rejecting										0.020	1.375	1.021
	Autonomous										−0.119	25.357	0.888**
	Controlling										0.024	0.906	1.024

Indices	Model 0	Model 1	Model 2	Model 3	Model 4
−2LL	692.345	639.328	575.267	533.313	495.285
Nag *R*^2^	N/A	0.011	0.181	0.280	0.363
Step *χ*^2^/*p*	N/A	3.728/0.589	64.1/<0.01	41.9/<0.01	38.0/<0.01
Model *χ*^2^/*p*	N/A	3.728/0.589	67.8/<0.01	109.7/<0.01	147.8/<0.01
Class Accur	54.8	56.7	65.7	70.2	72.8

−2LL, −2 log likelihood; Nag R^2^, Nagelkerke’s R^2^; Class Accur, classification accuracy; Dependent variable, internet gaming disorder; Model 1, demographic factors; Model 2, Model 1 + game play pattern; Model 3, Model 2 + Psychological status; Model 4, Model 3 + parenting style; K-ARS, Korean version of Dupaul’s ADHD rating scale; BIS/BAS, behavioral inhibitory system/behavioral activation system; K-WAIS-IV, Korean-Wechsler adult intelligence scale-IV; YIAS, young internet addiction scale; BDI-II, Beck depression inventory II; BAI, Beck anxiety inventory. *p < 0.05 and **p < 0.01.

### Testing for mediation

With the results of the hierarchical logistic regression analysis, attention problems, affective parenting styles, and autonomous parenting styles were determined to be significant predictive factors for IGD. With those factors, we performed tests for meditation.

**Figure 1:**
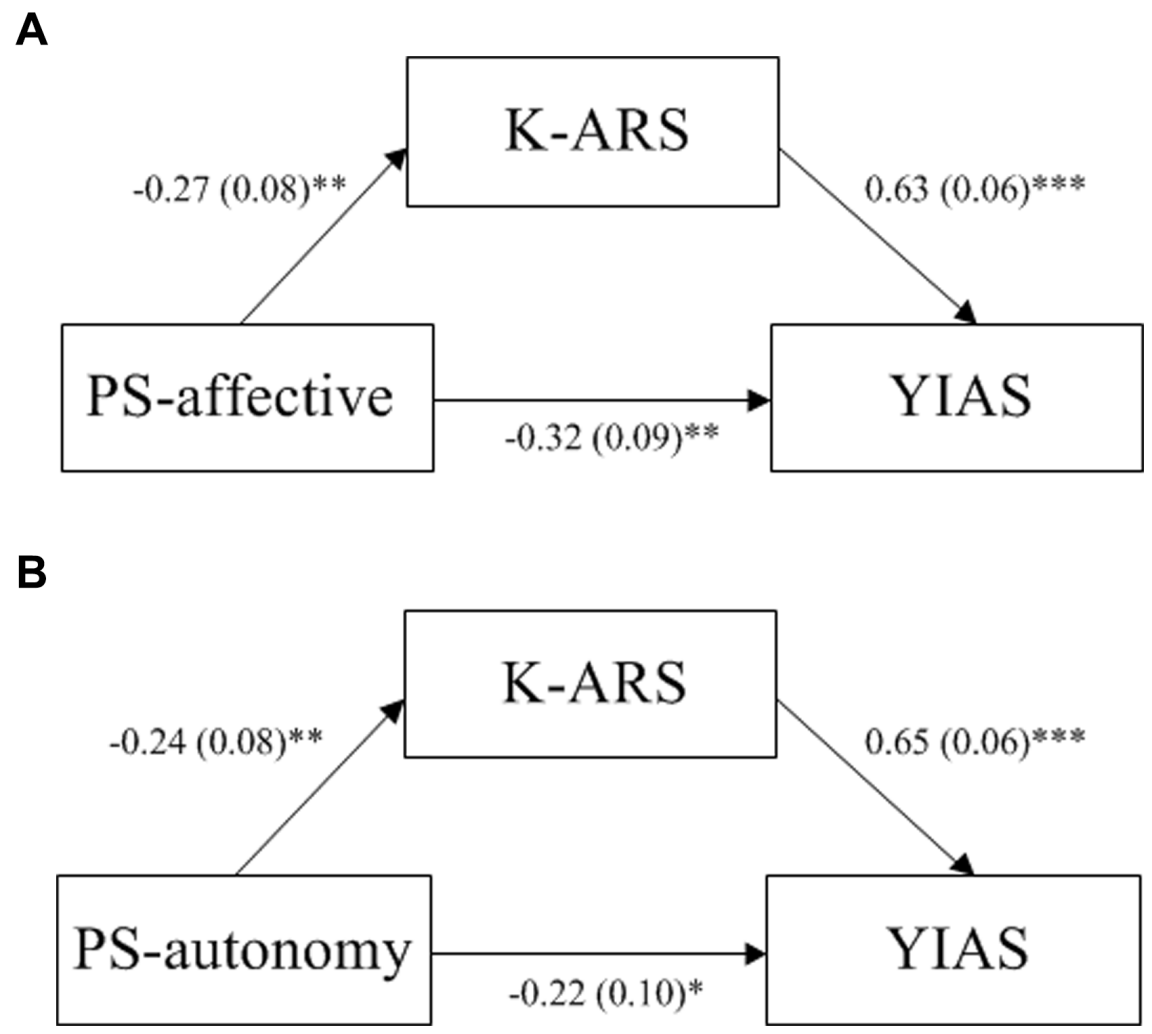
Mediation effect of attention problems on the link between parenting style and the severity of internet gaming disorder *p < 0.05, **p < 0.01, ***p < 0.001 **(A)** The link between an affective parenting style (PS-affective) and the severity internet gaming disorder (IGD) as assessed with the Young Internet Addiction Scale (YIAS) is mediated by attention as assessed using the Attention Deficit Hyperactivity Disorder Rating Scale (Korean version) (K-ARS). Path values are the path coefficients (standard errors). Direct effect = −0.32, SE = 0.09, 95% CI (−0.435 to −0.039). Indirect effect = −0.17, SE = 0.07, 95% CI (−0.317 to −0.023). **(B)** The link between an autonomous parenting style (PS-autonomous) and the severity internet gaming disorder (IGD) as assessed with the Young Internet Addiction Scale (YIAS) is mediated by attention as assessed using the Attention Deficit Hyperactivity Disorder Rating Scale-Korean version (K-ARS). Path values are the path coefficients (standard errors). Direct effect = −0.22, SE = 0.10, 95% CI (−0.437 to −0.061). Indirect effect = −0.16, SE = 0.07, 95% CI (−0.301 to −0.019).

Controlling for all the covariates, the scores for affective parenting style were negatively associated with the K-ARS scores (*β* = −0.271, *p* < 0.01), which in turn affected IGD (*β* = 0.630, *p* < 0.001). In addition, a significant residual direct effect was observed (*β* = −0.321, *p* < 0.01). This indicates that the K-ARS scores partially mediated the link between an affective parenting style and IGD (indirect effect = −0.171, SE = 0.074, 95%CI = −0.317 to −0.023; Figure 1).

Controlling for all the covariates, the scores for autonomous parenting style were negatively associated with the K-ARS scores (*β* = −0.244, *p* < 0.01), which in turn affected IGD (*β* = 0.645, *p* < 0.001). Furthermore, a significant residual direct effect was observed (*β* = −0.218, *p* < 0.05). This indicates that the K-ARS scores partially mediated the link between an autonomous parenting style and IGD (indirect effect = −0.157, SE = 0.072, 95%CI = −0.301 to −0.019; Figure 1).

## Discussion

In the current results, there were significant differences in the game play patterns, psychological status, and parenting styles between the IGD and healthy game play groups. In particular, the K-ARS scores, affective parenting styles, and autonomous parenting styles were significant predictive factors for IGD. In addition, attention problems could mediate the association between parenting style and the severity of IGD.

### Comparison of the game play patterns, psychological status, and parenting styles between the IGD and healthy game play groups

In current study, the IGD group showed increased BDI-II, BAI, K-ARS, and BISBAS scores compared with the healthy game play group. Many studies have already suggested that IGD is associated with various comorbidities including MDD, anxiety disorders, ADHD, and impulse control disorders (41–43). These psychiatric comorbidities are associated with the cause of IGD (44) and aggravate the progression of IGD (24). Of several comorbidities, ADHD was the most common comorbidity in the IGD group (26). Lee et al. (26) reported that an IGD group with a comorbidity of ADHD showed a poor clinical course of IGD and that changes in ADHD symptoms were associated with changes in IGD symptoms. In the hierarchical regression analysis in the current study, problematic attention scores were found to predict IGD.

In the current study, the scores for maternal affective attitudes and autonomous attitudes in the IGD group were significantly decreased compared with those observed in the healthy game play group. Several studies have suggested that parenting style influences psychopathology and the severity of IGD in children and adolescents (45, 46). Durkee et al. (45) reported that low parental involvement could predict a high risk of internet addiction in children (46). In addition, a negative parenting style including strict attitudes, heavy punishment, and low affection was found to provoke internet addiction in middle school and college students (27). However, previous studies of the relationships between parenting style and internet addiction (or IGD) have suggested that negative parenting is associated with IGD but that positive parenting has controversial protective effects against IGD in children and adolescents (47).

### Mediation effects of attention problems on the interaction between parenting style and IGD

As mentioned above, positive parenting may have protective effects against IGD in children and adolescents, but these remain controversial. However, in the current study, positive parenting styles including affective and autonomous parenting styles directly affected the severity of IGD. Consistent with the findings of previous studies (48–50), a less authoritative parenting style was found to be associated with IGD. On the other hand, there is controversy regarding the autonomous parenting style because both authoritative and autonomous styles share the characteristic of autonomy. Therefore, it is believed that the low level of autonomy characteristic of the autonomous parenting style may affect the severity of IGD. In contrast, in the current study, no association was found between the rejecting parenting style or controlling parenting style and IGD. Previous studies have indicated that the relationship between parenting style and IGD may vary depending on factors such as the age of the children (51). Specifically, whether the rejecting or controlling parenting style is associated with IGD remains disputed across studies. Therefore, the disparities in the findings of the literature may be attributed to differences in sample characteristics or the influence of parents with diverse cultural dynamics (51).

In the current study, attention scores were found to directly and indirectly mediate the relationship between affective attitudes and autonomous parenting styles and the severity of IGD. Several studies have suggested the mediation effects of ADHD symptoms on the risk of IGD in children and adolescents (52, 53). Jung et al. (52) reported that inattentive ADHD symptoms partially mediated the link between immersive tendencies and susceptibility to IGD. Lim et al. (53) showed that psychological status including anxiety, depression, and attention problems had a partial mediating effect on the link between aggression and the risk of IGD in 714 middle school students. It is possible that there is a bidirectional relationship between ADHD and IGD, in which the symptoms of ADHD contribute to an increased attraction to playing computer games and video games, while gaming, in turn, can worsen the symptoms of ADHD by reinforcing them. These symptoms may include inattention, disinhibition, impulsive response, and a strong desire for immediate rewards. In accordance with this, internet games have also been found to be used as a means of self-medication by children with ADHD (54).

Many studies have supported the relationships between affective and autonomous parenting styles and improving ADHD symptoms and mother–child relationships (55–57). Nelson et al. (56) reported a mediating effect of hyperactivity symptoms of ADHD on the relationship between mother and children. More severe hyperactivity symptoms of children at 5.5 years old was found to lead to increased maternal hostility to their children at 10 years old; in turn, this caused increased delinquent behaviors and aggression in adolescents (56). Thomassin and Suveg (57) also reported that parental support of autonomy moderated the link between ADHD symptoms and task perseverance in difficult puzzle tasks. Furthermore, Breaux et al. (55) suggested that an affective parenting style could facilitate the development of emotional regulation skills in children with ADHD.

### Limitations

There were several limitations in the current study. First, data on parenting style were gathered from the participants’ mothers. The father’s parenting style is also known to play an important role in children’s behavior (58). Second, the cross-sectional design of current study could not show causality or directionality. Finally, with the exception of the K-WISC-IV, most of the scales used in this study relied on self-report measures, which could have introduced bias. Future studies should recruit fathers to assess parenting style as well as apply a longitudinal design to delineate the directionality of the relationships between parenting style and IGD.

## Conclusion

The IGD group showed differences in K-ARS scores, affective parenting styles, and autonomous parenting styles compared with the healthy game play group. Moreover, attention problems were found to directly and indirectly mediate the relationship between positive parenting styles including affective and autonomous styles and the severity of IGD. Improved concentration through ADHD treatment could potentially help control the symptoms of IGD, making this treatment plan applicable in a clinical setting. In addition, providing parents with information about parenting styles that include emotional support may help to reduce the symptoms of both IGD and ADHD in children. In the future, our findings have the potential to aid in the development of educational resources regarding parenting styles.

## Data availability statement

The original contributions presented in the study are included in the article/supplementary material, further inquiries can be directed to the corresponding author.

## Ethics statement

The studies involving human participants were reviewed and approved by Chung Ang University Institutional Review Board. Written informed consent to participate in this study was provided by the participants’ legal guardian/next of kin.

## Author contributions

DH and SB designed the study and wrote the protocol. JL and HH conducted the statistical analysis. SC and HK wrote the manuscript. All authors contributed to the article and approved the submitted version.

## Funding

This research was funded by Ministry of Culture, Sports and Tourism and Korea Creative Con-tent Agency (Project number: R2020040186).

## Conflict of interest

The authors declare that the research was conducted in the absence of any commercial or financial relationships that could be construed as a potential conflict of interest.

## Publisher’s note

All claims expressed in this article are solely those of the authors and do not necessarily represent those of their affiliated organizations, or those of the publisher, the editors and the reviewers. Any product that may be evaluated in this article, or claim that may be made by its manufacturer, is not guaranteed or endorsed by the publisher.
